# Internet based discussion group

**Published:** 2010

**Authors:** Mukund Jagganathan

**Affiliations:** Department of Plastic Surgery, LTMG Hospital, Sion, Mumbai, India

Internet-based discussion groups are burgeoning up in most developed and developing countries. Japan itself has over 2 million posts per day on one particular forum[1] (http://en.wikipedia.org/wiki/Internet_forum). The purpose of a forum can be varied. Some blog sites, which also serve as discussion areas, are merely for the purpose of bashing concepts, ideas, and people.

Forums for educational or scientific discussions are very useful as they place several people, ideas, and suggestions on a common platform. As the cross-section of members is varied and often transcends geographic, ethnic, and ideological boundaries, there are likely to be multifactorial suggestions, which will usually present the solution in multiple options, one of which is likely to fit the bill.

However, there necessarily have to be some restraints, filters, and censoring to ensure that the site is not misused or abused. Individual grievances or witch hunts are detrimental and should be severely discouraged.

Let us look at some of the requirements, pluses, and limitations of such a discussion group.

## REQUIREMENTS

1. Moderator(s): This is one of the prime needs. The moderator should be proactive, but should be very clear in what goes in as a general post. This is probably the most difficult job. Frequently, there will be accusations of bias and one-upmanship, but he/she should exercise firm judgement. If for any reason a post is rejected, the reason should be explained confidentially to the concerned member. Similarly, if the moderator feels that a particular suggestion has already been posted earlier by a member, there is no need to post an identical message merely because it is given by another member. This will avoid unnecessary mails cluttering the inbox. The moderator also needs to keep a reasonable time limit for the discussion of a particular thread.
2. Members: Unfortunately, there is very little control over this. Mails introducing new members are not given enough attention. I feel that each new member must be vetted for genuineness and a general post informing that a new member has joined must be made by the moderator. There must also be a record of the member and the introducer so that in case of any issues, there is at least some degree of accountability. This is especially critical in a medical discussion forum that may have medicolegal ramifications. I feel that in the forum that has been highlighted in this article, not enough scrutiny is given to potential members.
3. Ethics and etiquette while posting a query:
  - If the patient’s face appears, or it is such a rare case that it can be recognized elsewhere, please take consent from the patient. Many patients are not comfortable if their case is discussed among several thousand members.
  - Please post genuine queries only, not something that has been operated and merely put up for the purpose of ascertaining whether someone else can come up with the same solution. It is better to put these cases in a separate heading called case discussion or review, not as a query.
  - Completion of a thread by posting the final results is very important. This is satisfying both for the person who posted the query and for the person who suggested a solution.
  - Personal communications frequently appear on the site, sometimes small thank you or congratulatory notes. While complimentary posts are okay, trivial ones should be sent to the person directly.
  - Images should be compressed to around 50–75 KB per image. Movies and big powerpoint presentations should be uploaded on the site, which can then be accessed.
4. Ethics and etiquette while answering a query:
  - If you have experience of similar cases, mention so. If not and, if you are suggesting what seems to be a logical answer, mention this as well. This will allow the original person to make a better judgment. Maybe he/she can read a little more about the case based on the inputs provided.
  - If another solution is not to your liking, do not trash it, but gently point out any shortcomings or reservations. If it is grossly wrong, and likely to actually cause harm to the patient (not very likely), send a personal mail to the original sender of the query. A good idea would be to send a copy to the person who suggested the solution so as to avoid any feelings of impropriety.
  - Please do not use this forum to solicit cases.
  - Please quote references only. Do not post the article in whole or part as it is not only unethical but may also violate several copyright issues.

## PLUSES

1. Identifying, discussing, and documenting solutions to potential problems are essential to the process of continuing medical education.
2. Many members do not have access to information as compared with others. This forum can help such members.
3. Many cases are unique and not covered in standard texts. These can be discussed in such forums.
4. Problems specific or peculiar to certain geographical, ethnic, or socioeconomic subsets can be better understood. This has been covered by the author and is very appropriate.

## LIMITATIONS

There are certain unanswered aspects of the implications of such group discussions:

1. In the event of a medicolegal issue, can this forum be quoted as a peer review and/or an opinion making body?
2. Somehow, if the patient gets access to this forum, it can be a disaster for the surgeon, especially if there is a complication of some sort. Again, this may hasten or add material to a lawsuit.
3. The author has covered the aspect of consent of the patient, and it is absolutely essential. As far as possible, the identity of the patient should be concealed.

In addition:

1. Unless the moderator is screening every mail, there can be unnecessary mails, repetitive mails as well as mails targeting specific people.
2. Other specialty people may “borrow” ideas and claim as their own.
3. Plagiarisation of photos and slides is very likely, despite attempts at watermarking.
4. Copyright articles displayed may invite legal action.

Notwithstanding these various shortcomings, an internet-based discussion forum is a very powerful tool for the benefit of most of its members. Careful and scrupulous adherence to clear-cut guidelines will avoid unnecessary ethical and legal problems. And, what about archiving? Are there plans to collate and archive posts and discussion threads for future reference? How do we envisage this as being practical, achievable and also secure?
